# The effect of motor control exercise versus placebo in patients with chronic low back pain [ACTRN012605000262606]

**DOI:** 10.1186/1471-2474-6-54

**Published:** 2005-11-04

**Authors:** Chris G Maher, Jane Latimer, Paul W Hodges, Kathryn M Refshauge, G Lorimer Moseley, Robert D Herbert, Leonardo OP Costa, James McAuley

**Affiliations:** 1 Back Pain Research Group, School of Physiotherapy, The University of Sydney, PO Box 170, Lidcombe, NSW, 1825, Australia; 2 Division of Physiotherapy, The University of Queensland, Brisbane Qld 4072, Australia; 3 Department of Human Anatomy & Genetics, Oxford University, South Parks Rd, Oxford, OX1 3QX, UK

## Abstract

**Background:**

While one in ten Australians suffer from chronic low back pain this condition remains extremely difficult to treat. Many contemporary treatments are of unknown value. One potentially useful therapy is the use of motor control exercise. This therapy has a biologically plausible effect, is readily available in primary care and it is of modest cost. However, to date, the efficacy of motor control exercise has not been established.

**Methods:**

This paper describes the protocol for a clinical trial comparing the effects of motor control exercise versus placebo in the treatment of chronic non-specific low back pain. One hundred and fifty-four participants will be randomly allocated to receive an 8-week program of motor control exercise or placebo (detuned short wave and detuned ultrasound). Measures of outcomes will be obtained at follow-up appointments at 2, 6 and 12 months after randomisation. The primary outcomes are: pain, global perceived effect and patient-generated measure of disability at 2 months and recurrence at 12 months.

**Discussion:**

This trial will be the first placebo-controlled trial of motor control exercise. The results will inform best practice for treating chronic low back pain and prevent its occurrence.

## Background

### The problem of chronic low back pain

Low back pain is the main cause of work absence and disability in industrialised societies. Approximately 10–20% of patients with low back pain develop chronic pain, defined as pain persisting for more than 3 months. Additional to their pain these patient's health problems typically include reduced physical function and psychological distress[1]. These patients use more than 80% of health care resources for back problems, and treatment has a low success rate [2].

In 2002, *arthritis and musculoskeletal disorders *were announced as the new National Health Priority Area in recognition of the major health and economic burden that these diseases place on the Australian community [3]. Amongst this group of diseases back pain is both the most prevalent and most costly single disease [4]. The 2001 National Health Survey revealed that *chronic back pain *is the most prevalent illness from the seven National Health Priority Areas [5].

The severity of chronic pain can be described with four hierarchical grades, Grades I–IV, that consider the pain intensity and the degree of disability associated with the pain [6]. An Australian population-based survey, noted that 22% of respondents reported chronic pain with 39% of respondents classed as Grade I (least severe), 35% as Grade II, 14% as Grade III and 13% as Grade IV (most severe) [7]. The most common cause of chronic pain was low back pain (45% of cases).

### Effectiveness of treatments for chronic low back pain

While there are a myriad of treatment options for chronic low back pain, there is only one clinical practice guideline for chronic non-specific low back pain: The European Guideline[8]. This guideline and the relevant Cochrane reviews [9] provide the most reliable sources of evidence on treatment for this condition. Unfortunately the Cochrane reviews provide fairly bleak reading for both clinicians and patients. Most of the reviews (7/13) concluded that the treatment under review was of unknown value. Five of the thirteen reviews concluded that there was some evidence for the treatment under review however significant limitations for each treatment were noted. These limitations included: no long term effect (e.g. back school); serious side effects (e.g. muscle relaxants); small effect size (e.g. massage); treatment improves outcomes other than pain (e.g. work conditioning) and no information available on patient or dose selection (e.g. behavioural treatment). The European Guideline produced similar conclusions [8]. In only one Cochrane review, the review of multidisciplinary rehabilitation/functional restoration, did the reviewers conclude that there was strong evidence for the therapy. However the reviewers also noted that these programs were only effective when they included >100 hours of therapy. Because these programs are multidisciplinary they are typically provided in a tertiary setting and because of the amount of time involved they are also very expensive. Accordingly functional restoration is usually reserved for the most severe cases of chronic low back pain.

The majority of patients with chronic low back pain has less severe pain (i.e. Grades I–III) and are typically managed in primary care. Not surprisingly clinicians find managing chronic low back pain difficult with qualitative research reporting that therapists' inability to identify effective treatment choices for their patients makes them state clinicians perhaps feel 'helpless' 'disillusioned' and 'pessimistic' [10]. Studies of patients reveal similar negative feelings and emotions [11].

To address this major problem, we plan to begin a coordinated program of research in which treatments that seem most promising are rigorously evaluated in randomised controlled trials. We define 'most promising treatments' as those that (i) appear to have clinically important effects that are maintained in the long term, (ii) are readily available and of modest cost and (iii) there is biological plausibility for the effect. Exercise therapy is our first candidate for evaluation in this program of research because it satisfies each of these three criteria, however at present trials have reported conflicting results.

While some trials of exercise therapy have reported large, durable and clinically important effects of treatment [12,13] others have not [14]. The uncertainty is reflected in the conclusion of the Cochrane review of exercise therapy: '...there is conflicting evidence on the effectiveness of exercise therapy...' [15]

Many factors are likely to have contributed to the inconsistent results across trials. Importantly, interpretation of the results of exercise trials is difficult because most trials have been pragmatic trials, comparing two active treatments delivered in routine practice (e.g. *exercise *vs. *usual medical care *[12]; *exercise *vs. *physiotherapy *[16]) These comparisons cannot provide a clear estimate of the effects of exercise treatment because most of the comparison treatments are also of unknown efficacy. Secondly, there has been insufficient appreciation by researchers conducting trials and by reviewers summarising trials of the wide variety of forms exercise can take and also trials do not control the quality of exercise intervention. While exercise is typically regarded as a single class of treatment we believe that this level of conception is inappropriate and analogous to not distinguishing between different classes and doses of drugs when prescribing medication. The types of exercise programs for chronic low back pain vary widely from land-based exercise versus exercise in water to isolated trunk exercise versus a walking program and it is unlikely that all programs are equally effective for all patients. Lastly, methodological quality varies greatly across previous exercise trials, for example in the Cochrane review [15] the least sound trial attended to none of the nine methodological criteria while the best attended to seven of the nine. Because methodological quality has been shown to affect the results of trials in other areas of health care [17] it is likely that a lack of rigor has contributed to the inconsistent results.

It is not sensible to talk about evaluating the efficacy of exercise without specifying the type of exercise. We have chosen to measure the efficacy of *motor control exercise *(sometimes called specific spinal stabilisation exercise) for chronic low back pain, rather than other forms of exercise, because it is a widely used form of exercise and there is an extensive body of literature that provides a rationale for the mechanism of action. The only way to clearly establish the value of motor control exercise in the management of chronic low back pain is to evaluate the efficacy of this form of exercise therapy in a methodologically sound randomised placebo-controlled trial. Prior to conducting a placebo-controlled trial of exercise we felt that it was prudent to identify the most promising form of exercise that would subsequently be evaluated in the placebo-controlled trial. To do this we conducted a randomised controlled trial where 160 patients were randomised to an 8 week program of either motor control exercise or general exercise[18].

The trial demonstrated that both programs were accompanied by large improvements in pain and disability. Motor control exercise produced significantly better outcomes in the short term, and there was a trend for motor control exercise to produce better outcomes at 6 month follow-up. Accordingly we have chosen to evaluate motor control exercise in the proposed trial. Our choice coincides with the research agenda set by the 2004 European Guideline: "The effectiveness of specific types of exercise therapy needs to be further evaluated. This includes the evaluation of spinal stabilisation exercises..." [8] p 7.

### Motor control exercise: treatment rationale

The use of motor control exercise is based on research that has shown that:

(i) People with low back pain have changes in the strategy for control of the trunk muscles in that activity of the deep muscles is impaired (delayed, less tonic) and these muscles are atrophied[19,20].

(ii) Although all muscles contribute to control of movement and stability of the spine, the deep muscles have a critical role for control of intervertebral motion [21-25], but with the potential advantage of allowing dynamic control of the spine.

(iii) Evidence that people with back pain tend to adopt a strategy for increased stiffness and stability at the expense of spinal function [26].

(iv) Non-resolution of changes in the deep muscle system is linked to recurrences of low back pain [27].

The evidence above underpins the primary aim of motor control exercise, which is to re-establish normal control of the deep spinal muscles, reducing the activity of more superficial muscles that tend to stiffen the spine and have increased activity in low back pain, and then maintain normal control during progressively more demanding physical and functional tasks[28].

The key feature of the motor control exercise approach is the training of the deep trunk muscles in isolation *before *progressing to demanding tasks that train coordination of the deep and the superficial trunk muscles [28]. However, unlike functional restoration approaches, training the deep trunk muscles in isolation from the superficial trunk muscles is difficult. In order to teach patients how to contract the deep muscles of the spine, in addition to clinical skills of palpation and observation [29] physiotherapists need to use technical devices such as pressure monitors, electromyography and ultrasound imaging to provide feedback to the patient.

The premise of the motor control approach is that simple functional exercise alone does not re-establish coordination of the trunk muscles. This premise is supported by the finding that the adaptation of these muscles to pain is still present following recovery from an episode of low back pain, when patients have returned to normal functional levels [19,20]. Furthermore, recent data confirm that coordination of the abdominal muscles can be restored with training of specific activation of the trunk muscles, but not a simple activation during a sit up task [30]. Notably, non-resolution of muscle dysfunction is associated with increased back pain recurrence [27]. Also, asymptomatic people with normal activity levels who are unable to perform a task that is thought to reflect voluntary activation of the deep trunk muscles, are ~6 times more likely to develop back pain than asymptomatic people who are able to perform the same task [31].

### Motor control exercise: level I and II evidence

At present there is no evidence for the efficacy of motor control exercise in the treatment of chronic low back pain. No systematic review of motor control exercise has been published, although one is being completed by our group. While the majority of trials (5 of 8) report that motor control exercise is effective in the management of chronic or recurrent low back pain most (7 of 8) have permitted co- intervention so that the contribution of motor control exercise is unclear. Additionally, all of these previous trials have used other treatments of unknown efficacy as the comparison intervention and so treatment efficacy cannot be measured. For example the earliest trial [12] reported that motor control exercise is more effective than usual medical care however this result provides an ambiguous estimate of treatment effectiveness because other trials have reported that sham physiotherapy treatments are more effective than usual medical care [32].

We will evaluate the efficacy of motor control exercise in a placebo-controlled randomised controlled trial. The results of our study will be invaluable for more efficacious evidence-based management of patients with non-specific chronic low back pain. Once efficacy is established, we will be able to progress to measuring whether there are additive or multiplicative effects of other treatments that are commonly administered as co-interventions with motor control exercise and thus to being able to make valid recommendations for their use.

## Methods

### Overview of research design

The study will be a randomised, blinded, placebo-controlled trial of a motor control exercise program for patients with chronic low back pain. The exercise program will consist of 12 individually supervised half-hour sessions over an 8-week period with treatment outcomes measured at 2 months, 6 months and one year.

### Hypotheses

(i) An 8-week motor control exercise program designed to restore control of the trunk muscles improves pain, disability and global perceived effect in participants with chronic low back pain at 2 months follow-up.

(ii) The improvements in pain, disability and global perceived effect following motor control exercise are maintained at 6 and 12 months follow-up.

(iii) At 12 month follow up recurrence is less in the motor control exercise group.

### Subject recruitment

A total of 154 participants will be recruited into the study. Participants will be screened for suitability for motor control exercise according to usual clinical practice. The screening instruments identify participants who are unsuitable for exercise management of their low back pain because of significant co- morbidity (serious spinal pathology, contraindication to exercise). A clinical assessment will identify patients who we expect would best be managed by a motor control exercise program rather than some other form of exercise or physiotherapy management.

### Screening

To screen for serious pathology, the physiotherapist will conduct a diagnostic triage [33]. Participants in whom serious spinal pathology is suspected will be excluded from the trial and referred to their medical practitioner for review. Potential participants will be screened for contraindications to exercise using the Physical Activity Readiness Questionnaire [34]. If a volunteer provides a positive response to items 1, 2, 3, 4, 6 or 7, the trial physiotherapist will discuss the case with the referring medical practitioner and if necessary a medical review will be undertaken to exclude any contraindication to exercise as listed in the ACSM guidelines [34].

The clinical assessment used to ensure that the motor control approach is indicated is based on the key text [28] and is a normal part of clinical assessment of low back pain. The assessment involves evaluation of the motor control strategy during a specific trunk muscle task – drawing in of the lower abdomen while maintaining an isometric contraction of the medial back muscles. The following criteria constitute correct performance of the task:

1. Moderate and sustained activation (> 10 seconds) of transversus abdominis

2. Moderate and sustained activation (> 10 seconds) of the lumbar multifidus muscles

3. Little or no activation of the global trunk muscles

4. No spinal or rib cage movement.

5. Normal breathing

Evaluation of task performance including satisfaction of the above criteria is dependent on the clinical skills of the physiotherapist. Patients who are unable to perform this task correctly will be considered suitable for motor control exercise.

Participants will be included if they meet all of the following inclusion criteria:

• Non-specific low back pain +/- leg pain of at least 3 months duration

• Currently seeking care for low back pain

• Aged greater than 18 and less than 80 years

• Comprehends English

• Clinical assessment indicates that the subject is suitable for motor control exercise

• Expects to continue residing in SWSAH region for study duration.

Participants will be excluded if they have any of the following:

• Suspected or confirmed serious spinal pathology (fracture, metastatic, inflammatory or infective diseases of the spine, cauda equina syndrome/widespread neurological disorder)

• Suspected or confirmed pregnancy

• Unable to speak English

• Nerve root compromise (2 of strength, reflex or sensation affected for same nerve root)

• Spinal surgery

• Scheduled for major surgery during treatment or follow-up period

• Any of the contraindications to exercise listed on page 42 of the ACSM guidelines [34]

• Any contraindication to pulsed ultrasound or pulsed shortwave.

Specific spinal pathology or contraindication to treatment may be suspected based on the results of the screening questionnaire and the Physical Activity Readiness Questionnaire. If the assessor suspects the presence of any pathology or contraindication to treatment, these subjects should be further investigated and medical clearance obtained, if necessary.

### Assessment and allocation

#### Outcome measures

Measures of outcomes will be obtained at follow-up appointments at 2, 6 and 12 months after randomisation. To maximise attendance at these follow-ups, appointments will be made by phone and then a letter will be sent confirming appointment and a reminder phone call will be made 24 hrs before the appointment. Every attempt (within ethical constraints) will be made to obtain outcome data, regardless of subject's compliance with trial protocols. Follow-up measures will be scored by an investigator who is blinded to group allocation. At 2 months, information about side effects of treatment will be collected from all participants using open-ended questioning.

Following the screening consultation, personal characteristics (age, gender, ethnicity, religion, weight, height, level of education, employment status, doctor's details and contact information) and information about symptoms of low back pain will be collected (eg DASS 21 [35]; Chronic Pain Grade Questionnaire) The following treatment efficacy variables will be measured at baseline, 2, 6 and 12 months.

1. Average pain intensity over last week (0–10 scale) [36-38]

2. Patient-generated measure of disability (Patient-Specific Functional Scale) [36-38]

3. Global perceived effect (Global Perceived Effect Scale) [36-38]

4. Condition-specific measure of disability (Roland Morris Disability Questionnaire) [36-38]

5. Recurrence at 12 months

The primary outcomes are pain, GPE and PSFS at 2 months and recurrence at 12 months.

#### Randomisation

Participants will be allocated to treatment group using sealed opaque envelopes. The allocation sequence will be generated by author CM. Participants will be scheduled to receive their first treatment within one week of randomisation.

#### Interventions

Contemporary physiotherapy practice in exercise prescription is to assess each patient and to implement the form of exercise that is most relevant to the particular clinical presentation. At present this widely accepted approach relies primarily upon the clinical expertise of the therapist. We have elected to evaluate motor control exercise delivered in this manner because this approach is regarded as contemporary best practice.

Participants in each group will receive 12 half hour treatments over an 8-week period, i.e. 2 sessions/week in the first month and 1 session/week in the second month. The treatment sessions are designed to become less frequent over time to encourage independence and continuation of exercise when therapy is complete. This is consistent with current clinical practice.

The *motor control exercise *program is based on the treatment approach reported by O'Sullivan et al [12], Richardson et al [28], and Moseley [39]. A brief description is provided below.

At the first session, participants will be comprehensively assessed and then will be prescribed exercises aimed at improving function of specific muscles of the low back region to be conducted in sessions 2–11. Stage 1 involves the most commonly prescribed exercise aimed at retraining multifidus (a back muscle) and transversus abdominus (a deep abdominal muscle); these exercises will be supplemented with exercises for the pelvic floor muscles, breathing control and control of spinal posture. Participants will be taught how to contract these muscles independently from the superficial trunk muscles [28,40]. Physiotherapists will use real-time ultrasound biofeedback to enhance learning of the tasks. When participants are able to perform these exercises, they will be gradually upgraded until the patient is able to maintain isolated contractions of these muscles for 10 seconds, up to a maximum of 10 repetitions, during normal respiration [28]. When this level of competence has been achieved, patients will be considered ready to progress to Stage 2.

Stage 2 of the approach involves increasing the complexity of the exercise by progressing through a range of functional tasks and exercises targeting coordination of trunk and limb movement and maintenance of trunk stability. The range and progression of exercises is well set-out in clinical texts [28] and is individualised to the patient based on this presentation. Participants require the ongoing support of a trained physiotherapist to ensure correct performance of the exercises. Session 12 is a discharge session where the patient's progress will be reviewed and patients will be prescribed exercises to continue at home.

The *placebo *intervention is 20 minutes of detuned short wave diathermy and 5 minutes of detuned ultrasound for 12 sessions over an eight week period. This attention control will be used because there is no known treatment effect from the detuned machines, but it has been established in previous trials (including one of our own [37]) that participants view this as a credible treatment. To increase the perceived credibility of the attention control, participants will undergo an examination including routine screening for contraindications at the first consultation and the normal clinical reassessment that would occur with the active forms of these interventions at each subsequent treatment. Each placebo treatment session will be 30 minutes in duration to match the active treatment sessions.

Participants in both treatment groups will be asked not to seek other treatments for their chronic low back pain and where possible not to change current medications during the treatment period. Several mechanisms will be used to ensure that the trial protocol is consistently applied. Protocol manuals will be developed and staff will be trained to ensure that screening, assessment, randomisation and treatment procedures are conducted according to protocol. To ensure standardisation across sites we will hold regular meetings with site visits and teleconferencing. An independent researcher will monitor a randomly chosen subset to ensure adherence to assessment, randomisation and treatment procedures.

If a participant is concerned about his or her condition during the study, the physiotherapist will screen for potentially serious pathology and, where appropriate, refer the patient to a medical practitioner. The medical practitioner will be asked not to request the participant's group allocation unless it is deemed necessary for medical care. At the completion of the exercise program, patients will be encouraged to continue the home exercise routine demonstrated at the discharge session. Participants will be free to seek other treatment after the experimental period.

After the first treatment session the patient will complete a treatment credibility scale [41]. At the 8 week follow-up information about side-effects of treatment will be collected using open-ended questioning. At the 12 month follow-up the participants will be asked to rate the helpfulness, understanding and friendliness of therapist and helpfulness of treatment and to nominate which treatment they thought they received. Additionally information about other treatment received for their low back pain during the study period will be sought using open-ended questioning.

#### Data integrity

The integrity of trial data will be monitored by regularly scrutinising data sheets for omissions and errors. Data will be double entered and the source of any inconsistencies will be explored and resolved.

### Data analysis

#### Treatment efficacy

In our primary analysis, we will use a regression model to test for the effect of treatment on outcome at 2, 6 and 12 months follow-up with the baseline value of the outcome entered as a covariate. A treatment effect size will be calculated for each of the follow-up time points and, if there is a statistically significant treatment effect at any time point, we will also calculate number needed to treat (NNT) to achieve pain recovery (pain < 1 out of 10: [42]) and 95% CI. The recurrence outcome will be analysed with logistic regression.

#### Predictor of response to treatment

We will include an interaction term baseline DASS-21 depression score × group to the regression analysis to see if the effect of motor control exercise is influenced by the baseline DASS-21 depression score.

### Sample size calculations

We have designed the study to detect a clinically important difference of 1 unit on the 0–10 pain intensity scale (estimate for SD = 2.00), 1 unit on the 0–10 patient specific functional scale (estimate for SD = 1.8); 1 unit on the 0–10 Global Perceived Effect Scale (estimate for SD = 1.65) and 4 units on the 24 item Roland Morris Disability Questionnaire (estimate for SD = 4.9). We have taken the SD estimates from a trial we completed that recruited a similar patient cohort[37] With specifications of alpha = 0.05, power = 0.80 a sample size of 77 participants per group is required to detect an effect size of 0.50 SD (the smallest effect size we have specified for the four outcomes). Based on the results of the same trial [37] we have allowed for 15% non-compliance with treatment, 15% loss to follow-up, and assumed a correlation between baseline and change scores of outcomes of 0.5. Accordingly we will recruit 77 participants per group or 154 participants in total.

### Justification of study design

#### Placebo

Designing an appropriate placebo treatment that mimics a physiotherapy exercise program is challenging. The sham interventions used in previous exercise trials do not satisfy the criteria of being both inert (e.g. the use of hot packs) and credible (e.g. allocation to a treatment waiting list). Accordingly, we will use sham electrotherapy as a control. This sham is clearly inert and is regarded as a credible treatment by participants. [37] To ensure that participants remain unaware of study group, it is necessary to carefully describe the study to patients. In the previous trial where we used sham electrotherapy as a control for exercise, we used the following description:

'In this trial normal physiotherapy treatment and placebo physiotherapy treatment will be provided. A placebo treatment is a harmless treatment delivered at less than the effective dose. We will not tell you which type of treatment you will receive and it is unlikely that you could distinguish them.'

Trial staff described the placebo intervention as 'pulsed ultrasound' and 'pulsed shortwave' and explained to patients that they would probably not feel any sensation during treatment.

#### Controlling bias

The trial has been designed to include key methodological features that have been recognised as minimising bias in clinical trials. These features include: true randomisation, concealed allocation, specification of eligibility criteria, blind outcome assessment, patient blinding, blind analysis and intention-to-treat analysis. The nature of the treatments precludes blinding of treatment provider. Trial staff will be trained to ensure consistency of screening, assessment, randomisation and treatment procedures. Participant's perception of the credibility of treatment will be determined after the first treatment [41]; and at the completion of treatment both assessors and participants will be asked to identify what treatment they think the participant received.

#### Outcomes

Measures of pain symptoms, disability and generic health status will be taken from the 'core set' of outcome measures for clinical research recently advocated by an international panel of back pain researchers [43]. The panel considered factors such as reliability, validity and responsiveness before recommending a measure. We have supplemented the back-related disability measure advocated in the core set (Roland Morris) with a patient-generated measure of disability (Patient-Specific Functional Scale) because there is evidence that patient-generated measures of disability are more responsive than condition-specific measures [37,44].

## Conclusion

We have presented the rationale and design of a randomized controlled clinical trial evaluating the effect of motor control exercise versus placebo in patients with chronic LBP. The results of this trial will be published as soon as they are available.

## Competing interests

All author(s) declare that they have no competing interests.

## Authors' contributions

CGM, JL, PWH, KMR, GLM, RDH and LOPC were responsible for the design of the study. LOPC and JM will act as trial coordinators. All authors have read and approved the final manuscript.

## Pre-publication history

The pre-publication history for this paper can be accessed here:


